# Integrated Experimental and Theoretical Studies on an Electrochemical Immunosensor

**DOI:** 10.3390/bios10100144

**Published:** 2020-10-17

**Authors:** Neda Rafat, Paul Satoh, Scott Calabrese Barton, Robert Mark Worden

**Affiliations:** 1 Department of Chemical Engineering and Materials Science, Michigan State University, 428 S. Shaw Lane, East Lansing, MI 48824, USA; rafatned@msu.edu (N.R.); satoh@msu.edu (P.S.); scb@msu.edu (S.C.B.); 2 The Institute for Quantitative Health Science and Engineering, Michigan State University, 775 Woodlot Dr, East Lansing, MI 48824, USA; 3 Department of Biomedical Engineering, Michigan State University, 775 Woodlot Dr, East Lansing, MI 48824, USA

**Keywords:** immunosensor, amperometric biosensor, horseradish peroxidase, antibody, catechol, mathematical model, flux control, dimensionless, design of experiments

## Abstract

Electrochemical immunosensors (EIs) integrate biorecognition molecules (e.g., antibodies) with redox enzymes (e.g., horseradish peroxidase) to combine the advantages of immunoassays (high sensitivity and selectivity) with those of electrochemical biosensors (quantitative electrical signal). However, the complex network of mass-transfer, catalysis, and electrochemical reaction steps that produce the electrical signal makes the design and optimization of EI systems challenging. This paper presents an integrated experimental and modeling framework to address this challenge. The framework includes (1) a mechanistic mathematical model that describes the rate of key mass-transfer and reaction steps; (2) a statistical-design-of-experiments study to optimize operating conditions and validate the mechanistic model; and (3) a novel dimensional analysis to assess the degree to which individual mass-transfer and reaction steps limit the EI’s signal amplitude and sensitivity. The validated mechanistic model was able to predict the effect of four independent variables (working electrode overpotential, pH, and concentrations of catechol and hydrogen peroxide) on the EI’s signal magnitude. The model was then used to calculate dimensionless groups, including Damkohler numbers, novel current-control coefficients, and sensitivity-control coefficients that indicated the extent to which the individual mass-transfer or reaction steps limited the EI’s signal amplitude and sensitivity.

## 1. Introduction

Electrochemical biosensors are analytical devices that detect analytes by transforming a biochemical reaction into a quantitative, electrical signal. This class of biosensors has proven valuable in research, quality control, food safety, medical diagnosis, and monitoring of therapeutic efficacy [1]. Miniaturized amperometric biosensors that use redox enzymes to generate an electric current in response to voltage applied at a working electrode have been successfully commercialized; personalized blood glucose meters used by diabetics represented 85% of the total biosensor market in 2008 [2]. By 2013, the worldwide market for glucose-monitoring biosensor systems was estimated to be billions of dollars per year, with screen-printed-electrode (SPE) arrays that served as single-use biosensor “strips” representing two thirds of that market [3]. The disposable, redox-enzyme-based biosensor market is being further expanded via the commercialization of glucose-monitoring systems for animals [4].

Immunoassays based on the exceptionally high binding selectivity and affinity of biological recognition molecules (predominantly antibodies, but also aptamers [5]) have a projected global market that is expected to reach $8 billion in 2022 [6]. Immunoassays typically involve a “sandwich” molecular architecture, in which immobilized capture antibodies first bind target-analyte molecules present in the liquid sample, and then secondary antibodies labeled with reporter molecules that generate an optical signal also bind the analyte molecules. The resulting molecular “sandwiches” consist of an analyte molecule held between primary and secondary antibody molecules. To estimate the analyte concentration, the surface concentration of bound reporter molecules is measured by the intensity of the optical signal they generate, and a calibration curve is used to convert the reporter molecule’s concentration into the analyte concentration [7]. Commonly used reporter molecules for immunoassays include redox enzymes whose products can be measured optically, such as horseradish peroxidase (HRP). HRP offers multiple advantages as a reporter. It is robust, has a relatively small molecular size, is inexpensive, is readily bound to antibodies in an active form, has a high turnover rate, and can oxidize a wide range of substrates to yield optically active products [8,9].

Whereas virtually all commercial immunoassay systems involve optical detection, the benefits of integrating electrochemical biosensors and immunoassays have been recognized [10]. Such hybrid electrochemical immunosensors (EI) have the potential to combine the advantages of immunoassays (extremely high sensitivity and selectivity) with those of electrochemical biosensors (reproducible, quantitative, continuous electrical output). The electrical output is achieved by forming a sandwich molecular architecture on the working electrode, and the reporter molecule triggers an electrical signal. Redox enzymes are commonly used as EI reporters, because some of their reaction products can be either oxidized or reduced at the working electrode, resulting in an electric current that serves as the EI’s output. This approach offers exceptional versatility, because an EI biosensor could be developed for virtually any analyte for which antibodies can be developed. Also, inexpensive, disposable SPE arrays designed to be read by portable meters similar to glucose meters EI could be mass-produced. The resulting EI platform would enable an extremely wide range of molecular and cellular analytes to be accurately measured with high sensitivity and selectivity, ease of use, low cost, and portability [11,12,13,14,15]. Prototype EI systems have been developed for healthcare applications. Sanchez-Tirado et al. fabricated an EI to measure cytokines used as markers of inflammation [16]. Tallapragada et al. developed an EI for human epidermal growth factor receptor 2 (HER2) that had a detection limit of 4 ng/mL [17]. Dempsey et al. described a disposable, printed lateral flow EI for human cardiac troponin T (cTnT) [18]. The reporter used in all of these studies, HRP, generated an oxidized product that was electrochemically reduced at the working electrode, resulting in a continuous amperometric output.

However, commercial implementation of EI systems has been hampered by the complexity of the multiple molecular mass-transfer, binding, and reaction steps that give rise to the electrical signal. This complexity complicates efforts to design new EIs that achieve specified performance metrics, including the lower detection limit and sensitivity (defined as change in output per unit change in analyte concentration). Fabrication methods and operating conditions needed to achieve these metrics are expected to vary between EI systems, due to factors including analyte-antibody binding affinities, the concentrations of primary antibodies bound to the electrode, and the kinetics of both the reporter enzyme’s reaction and the electrochemical reaction. These kinetics will, in turn, be influenced by the liquid sample’s properties, including its pH, its concentrations of the analyte, and substrates for the enzymatic reaction. Moreover, the concentrations of redox-active interferents in the sample may limit the working electrode’ voltage.

Development of robust product-design algorithms for new EI systems that meet specified performance metrics would be aided by mechanistic mathematical models that quantitatively describe the rates of the key molecular mass-transfer, binding, and reaction steps. Such models would enable the step(s) that limits performance to be identified and guide strategies to overcome such limitation(s). To date, few mechanistic models of HRP-based EIs have been reported [19,20,21,22,23], and these models have not been sufficiently comprehensive to predict how the output would vary with key independent variables, including the working electrode’s applied voltage (*E*), the pH, and the concentrations of HRP’s substrates. Such models are needed to help design EIs, identify factors that limit their performance properties, and guide research strategies to optimize EI systems. Mechanistic models would also help support petitions for U.S. Food and Drug Administration (FDA) approval of EI systems for healthcare applications. The FDA requires that stringent accuracy and consistency standards be met by portable glucose monitoring systems while in the hands of lay users [24], and similar requirements would be expected for EIs. Mechanistic models would enable rapid, in-silico hypothesis testing, including “what-if” studies to assess whether non-standard use by lay users could result in dangerously incorrect readings.

This paper addresses the need for such mechanistic models by presenting a novel, integrated experimental and mathematical framework to characterize EI performance, and then applies the framework to optimize performance of a novel EI that can detect a target protein (mouse IgG) at the ng/mL level. The framework includes three components. The first is a detailed mechanistic model that can predict the rates of the individual mass-transfer and reaction steps that give rise to the EI’s amperometric output. The second is a statistical-design-of-experiments approach that generates an empirical, statistical model describing the effects of key independent variables on the EI’s output. This statistical model is used both to optimize the EI system and to help validate the mechanistic model. The third is an integration of dimensional analysis with principles of flux-control theory to quantify the extent to which individual mass-transfer and reaction steps limit the EI’s sensitivity and output current (*J*). The paper concludes by discussing the utility of the integrated experimental and mathematical framework for future design, optimization, and validation of EI systems.

## 2. Materials and Methods

### 2.1. Materials

Thioctic acid, sodium phosphate (monobasic and dibasic), mouse IgG, anti-mouse IgG antibody (ap124), HRP-conjugated-goat anti-mouse IgG (a5278), TWEEN 20, *H*_2_*O*_2_), *C*, and N-hydroxysulosuccinimide sodium salt (NHS) were obtained from Sigma Aldrich (St. Louis, MO, USA). MES buffered saline packs and 1-ethyl-3-(3-dimethylaminopropyl carbodiimide hydrochloride) (EDC) were purchased from ThermoFisher Scientific (Waltham, MA, USA). Ultrapure water (18.2 MΩ) was produced by a Nanopure-UV four-stage purifier (Barnstead International, Dubuque, IA, USA); the purifier was equipped with a U.V. source and a final 0.2 μm filter. Ultrapure water was used in all aqueous solutions. Screen-printed electrodes were obtained from Conductive Technologies Inc. (York, PA, USA) and Metrohm DropSens (Oviedo, Asturias, Spain) (models DRP-250BT and DRP-110SWCNT).

### 2.2. Preparation of Immunosensing Layer

The immunosensing layer was prepared by using 1-ethyl-3-(3-dimethylaminopropyl (EDC) and N-hydroxysulfosuccinimide sodium salt (NHS) chemistry to attach the primary (capture) antibodies covalently to carboxylate groups present on the DropSens array’s working electrodes. EDC-NHS chemistry has been widely used to fabricate the immunosensing layers of EIs [25,26,27]. EDC is a zero-length cross-linker that activates carboxylate groups for covalent coupling to primary amines. The addition of NHS with EDC results in an NHS ester intermediate that reacts rapidly with primary amines, thereby increasing the efficiency of the coupling reaction [28]. Cleaned gold SPEs were dipped in 15 mM thioctic acid in ethanol for 1 h. The resulting carboxylated SPEs were washed with ethanol and dried under nitrogen. The carboxyl groups were activated by incubating the SPEs in 100 mM MES buffer containing 5.0 mM EDC and 9.0 mM NHS at pH 4.6 for 1 h at room temperature. Electrodes were then rinsed with MES buffer and dipped in 6 µg/mL goat anti-mouse IgG antibody in 50 mM phosphate buffer at pH 7 for 2 h. The primary-antibody-functionalized SPEs were then washed with phosphate buffer. To block nonspecific binding of the secondary antibody, the SPEs were incubated in 2 % bovine serum albumin (BSA) in phosphate buffer for 1 h at room temperature. The resulting functional SPEs were washed with phosphate buffer at pH 7 and stored in phosphate buffer at 4 °C.

**Figure 1:**
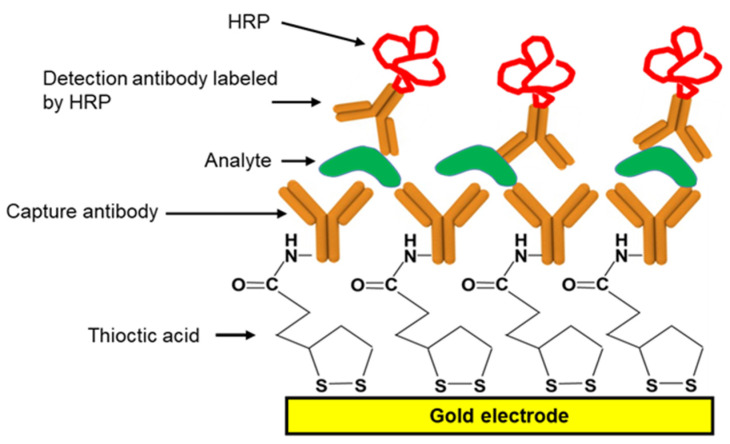
Schematic diagram of immunosensing layer showing molecular sandwiches containing the capture antibody, the target analyte, and the HRP-tagged secondary antibody bound to the EI’s gold working electrode.

SPEs were each dipped in a standard solution having a known concentration of the target analyte (mouse IgG) in a 2% aqueous BSA solution in 50 mM phosphate buffer at pH 7 for 1 h at room temperature. The SPEs were then washed four times with washing buffer (0.05% TWEEN20 in 50 mM phosphate buffer at pH 7) and incubated in a [1:333] dilution of HRP-conjugated-goat anti-mouse IgG in pH 7, 50 mM phosphate buffer in 2% BSA (Figure 1). After 1 h, the electrodes were rinsed four times with washing buffer and stored in phosphate buffer at 4 °C until the electrochemical measurements were conducted.

### 2.3. Electrochemical Measurement of EI Signal

The EIs were removed from the refrigerator and allowed to equilibrate at room temperature. Forty µL of a solution (subsequently referred to as the “bulk solution”) containing 50 mM phosphate buffer, 1 mM $H_{2}O_{2}$, and 8 mM *C* were added on the SPE. Wire leads from a potentiometer (CHI 660, C.H. Instruments, Austin, TX, USA) were connected to the EI’s working, reference, and auxiliary electrodes, and a reduction potential of −0.2 V relative to an Ag/AgCl reference electrode was applied to the working electrode. After about 1 min, the reduction current (i.e., the EI’s signal (*J*)) reached a steady-state value, and the current level was recorded as the EI’s output for that set of experimental conditions. Each EI was used once. All electrochemical potentials given in this paper are relative to an Ag/AgCl reference electrode.

### 2.4. Optimization of EI Operating Conditions and Characterization of EI Performance Properties

A statistical design of experiment (DOE) approach was used for two purposes: (1) to determine the values of key independent variables that optimized the EI’s signal and (2) to obtain an empirical equation that described the effects of the key independent variables on the EI’s signal to help validate the mechanistic model. The independent variables expected to most strongly affect the performance of the EI described above included (1) the working electrode’s *E*, (2) the bulk solution’s [*C*], (3) the bulk solution’s [*H*_2_*O*_2_], and (4) the bulk solution’s pH [29].

A two-level half-factorial design with center points and three replicates for each experiment was set up using Minitab^®^ software (Supplementary Table S1). For each factor, the following three levels, denoted low (−1), center point (0), and high (+1), were chosen: −0.05 V, −0.125 V, and −0.2 V for *E*; 1.0 mM, 4.5 mM, and 9.0 mM for [*C*]; 0.5 mM, 1 mM, and 1.5 mM for $\left[H_{2}O_{2}\right]$; and 6.2, 6.6, and 7.0 for pH, respectively. To avoid electrical noise arising from reduction of redox-active interferents in the bulk solution [30], the lowest *E* value was set to −0.2 V. To control the rate of *C* autoxidation [31], 8 mM was selected as the highest [*C*] value. Experiments were conducted in triplicate for each combination of factors specified by Minitab^®^ using a constant analyte concentration of 40 ng/mL mouse IgG. Each EI’s signal was calculated as the difference between the *J* measured first in the absence of analyte and then in the presence of the analyte. All signal data were input to Minitab^®^, which provided a statistical analysis of the results. The experimental conditions that Minitab^®^ indicated were optimal for the EI were used in subsequent experiments to characterize the EI’s performance properties. In these experiments, the EI signal was measured in triplicate for six concentrations of the analyte.

## 3. Mechanistic Mathematical Model

**Figure 2:**
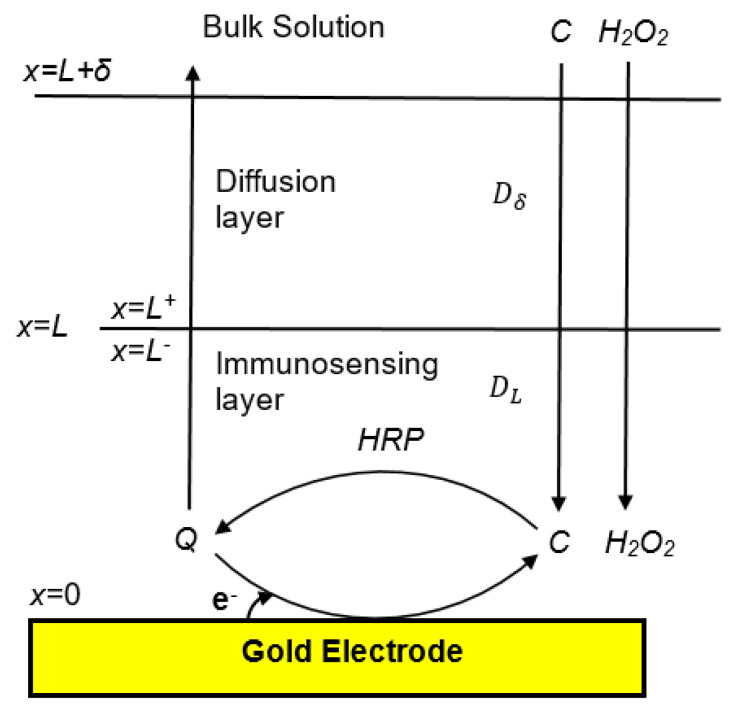
Schematic representation of diffusional mass-transfer, enzyme catalysis and electrochemical reaction steps happening on the biosensor interface.

The mechanistic mathematical model of the EI describes the transport and reaction processes involving catechol (C), O-quinone (Q), and hydrogen peroxide $\left(H_{2}O_{2}\right)$ that generate a current (*J*) at the EI’s working electrode. Differential mass-balance equations describe the diffusion of these species in the x direction (perpendicular to the electrode), through two layers (Figure 2) that lie between the electrode’s surface at *x* = 0 and the bulk solution: (1) the immunosensing layer between *x* = 0 and *x* = L containing the antibodies and HRP, and (2) a stagnant, aqueous, diffusion layer between *x* = L and *x* = L + *δ*. The HRP-catalyzed conversion of C and $H_{2}O_{2}$ to Q is assumed to occur uniformly throughout the immunosensing layer, and the electrochemical reduction of *Q* to *C* is assumed to occur on the electrode’s surface. The bulk solution is assumed be well-mixed, with the concentrations of all chemical species remaining constant at their initial values [32]. Mass transfer is assumed to follow Fick’s law, with a diffusion coefficient (*D*) that is assumed to be the same for Q, C, and $H_{2}O_{2}$ but to vary between the diffusion layer ($D_{\delta })$ and the immunosensing layer ($D_{L}$). The HRP concentration and maximum reaction rate constant ($V_{max}$) are assumed to be uniform throughout the immunosensing layer [33].

### 3.1. Kinetics of Enzymatic and Electrochemical Reactions

The non-linear, ping-pong kinetic mechanism describing HRP oxidation of *C* in the presence of $H_{2}O_{2}$ is shown in Reactions (i)–(iii) [33,34,35,36]:

HRP (Fe^3+^) + H_2_O_2_ → Compound(I) + H_2_O (i)

Compound (I) + *C* → Compound (II) + *Q* (ii)

Compound (II) + *C* → HRP (Fe^3+^) + *Q* (iii)

where compounds (I) and (II) are oxidized intermediates of HRP. The kinetic formula resulting from this mechanism [21,37,38,39,40] is:

(1) $$V=\frac{V_{max}\left[H_{2}O_{2}\right]\left[C\right]}{K_{m}^{C}\left[H_{2}O_{2}\right]+K_{m}^{H_{2}O_{2}}\left[C\right]+\left[H_{2}O_{2}\right]\left[C\right]}$$

where V is the reaction rate, $V_{max}$ is the maximum reaction rate constant ($V_{max}=k^{cat\;}\left[HRP\right])$, $k^{cat\;}$and [HRP] are turnover number and HRP concentration within the immunosensing layer, respectively; $K_{m}^{C}$ and $K_{m}^{H_{2}O_{2}}$ are the corresponding Michaelis–Menten constants, and $\left[H_{2}O_{2}\right]\;$and [C] are $H_{2}O_{2}$ and *C* concentrations, respectively.

Molecules of *Q* produced by HRP can be reduced back to *C* at the surface of the working electrode in a two-electron, two proton reaction shown in Reaction (iv) at a rate described by the Butler–Volmer Equation (2) [41]:

*Q* + 2e^−^ + 2H^+^ → *C* (iv)

(2) $$J=nFD_{L}{\left[\frac{dQ}{dx}\right]}_{x=0}=\;nFK_{0}{\left[Q\right]}_{x=0}\;e^{\left(-\frac{\alpha nF\left(E-E_{h}\right)}{RT}\right)}-nFK_{0}{\left[C\right]}_{x=0}\;e^{\left(\frac{\left(1-\alpha \right)nF\left(E-E_{h}\right)}{RT}\right)}$$

where, J is the electric current density, n is the number of transferred electrons (*n* = 2 for this reaction), α is the charge transfer coefficient (assumed 0.35), F is the Faraday constant (96,485 C mol^−1^), $K_{0}$ is the apparent electron transfer rate constant for *Q*, R is the universal gas constant (8.314 J K^−1^ mol^−1^), T is the absolute temperature (298 K), and $E_{h}$ is the redox potential for electrochemical reduction of *Q* to *C* under the experimental conditions used (0.15 V at pH 6.2). Values of $E_{h}$ for a given set of experimental conditions were determined as the midpoint potential ($E_{mid})$ between the cathodic peak (for *Q* reduction) and anodic peak (for *C* oxidation) of cyclic voltammograms obtained under the same conditions [42]. The calculated value of *J* was taken to be the current generated by the EI.

The effect of pH on $E_{mid}$ is shown in Equation (3) [43,44], in which *m* (=2) and *n* (=2) are the number of protons and electrons involved in the reduction of *Q*, respectively. This equation indicates that increasing the pH would make $E_{mid}$ more negative and thereby reduce the working electrode’s overpotential, reaction rate, and EI’s signal, according to the Butler–Volmer equation. To simulate the effect of pH on$\;E_{h}$, Equation (3) was incorporated in the mechanistic model.

(3) $$E_{mid}\;\sim \;\mathrm{const}-2.303\frac{\mathrm{mRT}}{\mathrm{nF}}pH$$

### 3.2. Mass Balance Equations

Assuming one-dimensional diffusion in the *x*-direction, the steady-state, differential, mass balance equations including diffusion and enzymatic reaction for$\;H_{2}O_{2}$, C, and *Q* across the immunosensing layer (0 < *x* < L) are shown in Equations (4)–(6) [33,45,46,47]:

(4) $$0=D_{L}\frac{d^{2}\left[H_{2}O_{2}\right]}{dx^{2}}-\frac{V_{max}\left[H_{2}O_{2}\right]\left[C\right]}{K_{m}^{C}\left[H_{2}O_{2}\right]+K_{m}^{H_{2}O_{2}}\left[C\right]+\left[H_{2}O_{2}\right]\left[C\right]}$$

(5) $$0=D_{L}\frac{d^{2}\left[C\right]}{dx^{2}}-\frac{V_{max}\left[H_{2}O_{2}\right]\left[C\right]}{K_{m}^{C}\left[H_{2}O_{2}\right]+K_{m}^{H_{2}O_{2}}\left[C\right]+\left[H_{2}O_{2}\right]\left[C\right]}$$

(6) $$0=D_{L}\frac{d^{2}\left[Q\right]}{dx^{2}}+\frac{V_{max}\left[H_{2}O_{2}\right]\left[C\right]}{K_{m}^{C}\left[H_{2}O_{2}\right]+K_{m}^{H_{2}O_{2}}\left[C\right]+\left[H_{2}O_{2}\right]\left[C\right]}$$

### 3.3. Boundary Conditions

Previous mathematical models [47,48,49,50] describing electrochemical reduction of *Q* have assumed the electrochemical driving force (*E-E_h_*) was sufficiently large that [*Q*] at the electrode’s surface (where *x* = 0) could be assumed to be approximately zero Equation (7).

(7) $${\left[Q\right]}_{x=0}=0$$

However, this assumption is likely to be invalid for an EI under some realistic operating conditions. For example, to avoid electrical noise and/or interference by electroactive species in the solution, it may be desirable to use a moderate (*E-E_h_*) value, for which ${\left[Q\right]}_{x=0}$ would not be vanishingly small, and use of Equation (7) would cause significant error in the model’s predictions. For that reason, we used the Butler-Volmer equation (Equation (2)) as a boundary condition at the working electrode surface. This equation is valid over the entire spectrum of positive and negative (*E-E_h_*) values.

Because *Q* reduction at the electrode generates *C* in equimolar amounts, the fluxes of *Q* and *C* at *x* = 0 were assumed to be equal in magnitude but opposite in sign (Equation (8)). Control experiments showed that *J* caused by the reduction of $H_{2}O_{2}\;$was close to zero under the experimental conditions (Figure S1, available in supplementary information). Therefore, the flux of $H_{2}O_{2}$ at *x* = 0 was assumed to be zero Equation (9).

(8) $$J=nFD_{L}{\left[\frac{dQ}{dx}\right]}_{x=0}=-nFD_{L}{\left[\frac{dC}{dx}\right]}_{x=0}$$

(9) $${\left[\frac{dH_{2}O_{2}}{dx}\right]}_{x=0}=0$$

Partitioning kinetics of all reactants between the diffusion layer and the immunosensing layer were assumed to be rapid enough that the interfacial concentrations were assumed to remain at equilibrium. Identical partition coefficients ($k_{p}$) were assumed for all reacting species Equations (10)–(12).

(10) $${\left[Q\right]}_{x=L-}=k_{p}{\left[Q\right]}_{x=L+}$$

(11) $${\left[H_{2}O_{2}\right]}_{x=L-}=k_{p}{\left[H_{2}O_{2}\right]}_{x=L+}$$

(12) $${\left[C\right]}_{x=L-}=k_{p}{\left[C\right]}_{x=L+}$$

The bulk solution (at x = ∞) was assumed to be well mixed and have the concentrations indicated in Equations (13A)–(13C).

(13A) $${\left[C\right]}_{x=\infty }=C\left(\infty \right)$$

(13B) $${\left[H_{2}O_{2}\right]}_{x=\infty }=H_{2}O_{2}\left(\infty \right)$$

(13C) $${\left[Q\right]}_{x=\infty }=0$$

No reaction is assumed to occur in the diffusion layer, so the mass transfer rate of $C,\;H_{2}O_{2}$ and *Q* across this layer is modeled as the product of a mass transfer coefficient (*D/δ*) and the concentration driving force across the layer. Also, at the interface between the diffusion layer and the immunosensing layer, the diffusive fluxes of $C,\;H_{2}O_{2}$ and *Q* exiting one layer are assumed to be equal to those entering the other layer Equations (14)–(16).

(14) $$D_{L}{\left[\frac{\partial Q}{\partial x}\right]}_{x=L-}=-\frac{D_{\delta }}{\;\delta }\left\{{\left[Q\right]}_{x=L+}-0\right\}$$

(15) $$D_{L}{\left[\frac{dH_{2}O_{2}}{dx}\right]}_{x=L-}=\frac{D_{\delta }}{\delta k_{p}\;}\left\{k_{p}\;H_{2}O_{2}\left(\infty \right)-{\left[H_{2}O_{2}\right]}_{x=L-}\right\}$$

(16) $$D_{L}{\left[\frac{dC}{dx}\right]}_{x=L-}=\frac{D_{\delta }}{\delta k_{p}\;}\left\{k_{p}\;C\left(\infty \right)-{\left[C\right]}_{x=L-}\right\}$$

The coupled, second-order differential Equations (4)–(6) that described nonlinear kinetics of HRP-catalyzed oxidation of *C* to *Q* Equation (1) and electrochemical reduction of *C* back to *Q* Equation (2), along with the boundary conditions Equations (8)–(16) were solved numerically using function BVP4C in MATLAB (codes available in supplementary information).

## 4. Results and Discussion

### 4.1. EI System’s Properties under Optimal Operating Conditions

**Figure 3:**
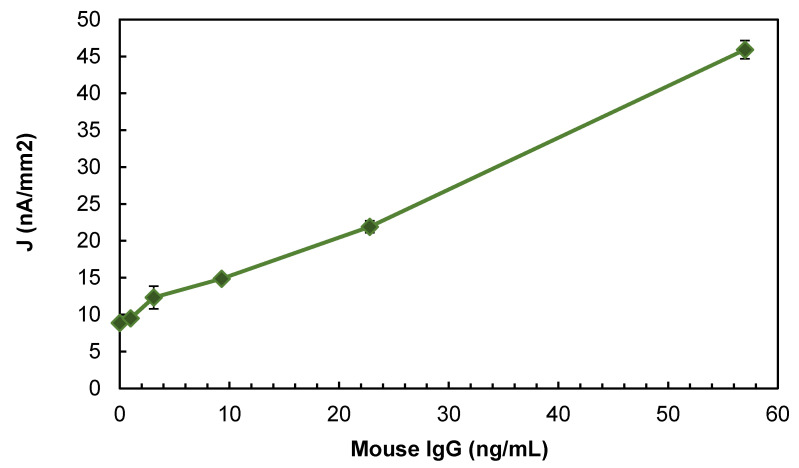
The dose response for mouse IgG on gold Dropsens SPEs under optimal experimental conditions. Error bars show ± standard deviation from the mean of 3 replicates. (Figure S2 in supplementary information compares the dose reponse before and after optimization of experimental conditions).

Based on the half-factorial experiments with a centerpoint, the experimental conditions that optimized the EI signal were *E* = −0.2 V, [*C*] = 8 mM, pH = 6.2, and $\left[H_{2}O_{2}\right]$ = 1 mM. The subsequent EI characterization experiments, which were conducted under these optimal experimental conditions (Figure 3), indicated that the EI’s limit of detection was 1 ng/mL, its sensitivity was 0.63 nA mL/(ng mm^2^), and its inter-assay/intra-assay variation was less than 5%.

### 4.2. Validation of Mechanistic Model

Minitab’s^®^ statistical analysis of the experimental optimization studies was integrated with the mechanistic mathematical model of EI operation for three purposes: (1) to help validate the mechanistic model, (2) to explain trends seen in the experimental data, and (3) to develop new methods to identify factors that limit an EI system’s signal strength and sensitivity to the target analyte.

Some of the constants used in the mechanistic model (Table 1) were obtained from literature data. Others were estimated by fitting the model to the empirical, statistical model that Minitab^®^ generated from the experimental optimization studies. The statistical model was a best-fit polynomial that expressed the EI’s signal as a function of the four factors. The polynomial had a linear term for each factor and binary, ternary, and quaternary product terms for each combination of factors to simulate interactions between factors (Equation (S1), available in supplementary information).

Values for the kinetic constants of HRP’s kinetic model were obtained from the BRENDA database [51]. The diffusion layer (δ) for the unstirred bulk solution was assumed to remain constant [52] at a value of 200 µm [53,54]. The thickness of immunosensing layer was assumed to be 25 nm [55]. Values of diffusion coefficients in the immunosensing layer and diffusion layer were assumed to be 2.5 × 10^−6^ cm^2^ s^−1^ and 2.2 × 10^−5^ cm^2^ s^−1^, respectively [56]. Values of $K_{0}$ and [HRP] (5.0 × 10^−7^ cm s^−1^ and 0.5 µM, respectively) were fit to the experimental data obtained using a constant analyte concentration of 40 ng/mL. Because the deposition of HRP molecules in the immunosensing layer results from formation of sandwich molecular architectures, the HRP value is expected to vary with the analyte concentration in the bulk liquid.

**Figure 4:**
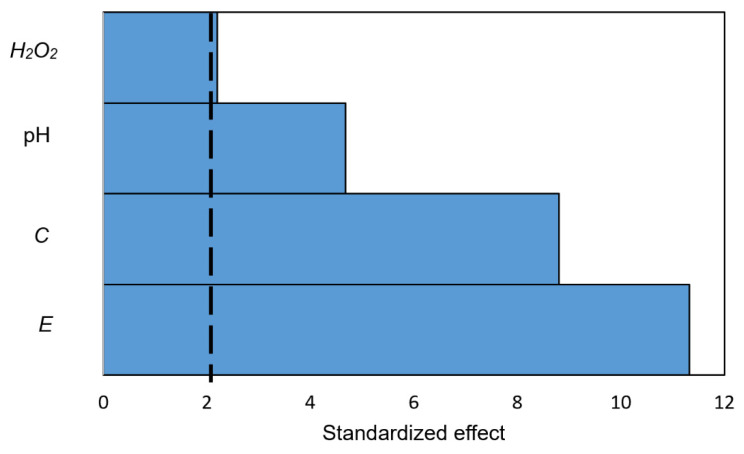
Pareto chart showing the standardized effect (SE) of factors *E*, [*C*], pH, and $[H_{2}O_{2}$] on biosensor signal. The terms with an SE value greater than the threshold value marked with the dotted line (SE = 2.09) exerted a statistically significant effect on biosensor signal at the 95% confidence level.

**Figure 5:**
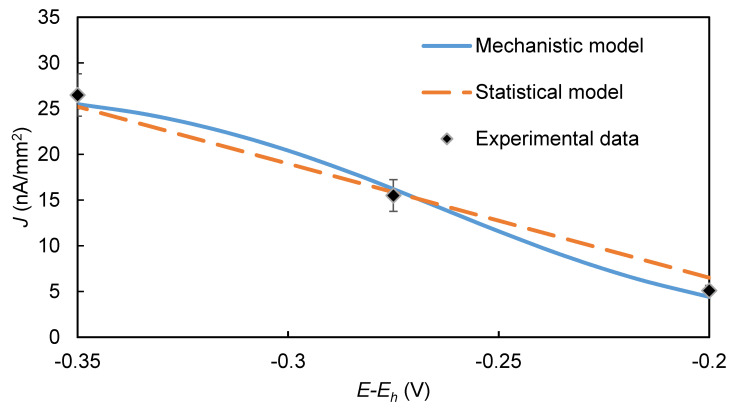
Effect of working electrode overpotential (*E-E_h_*) on the steady-state EI’s signal. [*C*] = 8 mM, $\left[H_{2}O_{2}\right]$ = 1 mM, pH = 6.2, [HRP] = 0.5 µM.

**Figure 6:**
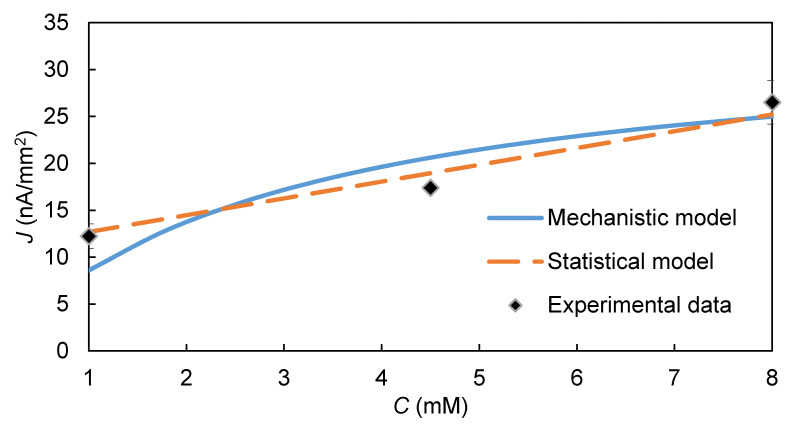
Effect of [C] on the steady-state EI’s signal: comparison of model prediction and experimental data. $\left[H_{2}O_{2}\right]$ = 1 mM, pH = 6.2, [HRP] = 0.5 µM, *E-E_h_* = −0.35 V.

**Figure 7:**
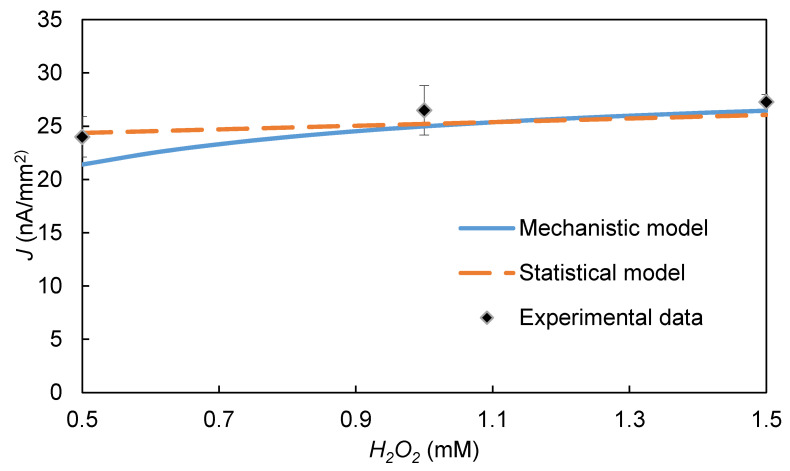
Effect of $\left[H_{2}O_{2}\right]$ on the steady-state EI’s signal: comparison of model prediction and experimental data. [*C*] = 8 mM, pH = 6.2, [HRP] = 0.5 µM, *E-E_h_* = −0.35 V.

**Figure 8:**
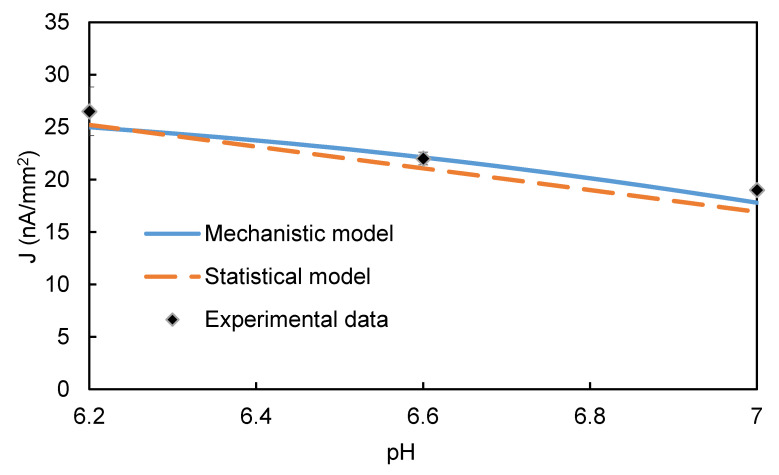
Simulation of pH effect on the steady-state EI’s signal. [*C*] = 8 mM, $\left[H_{2}O_{2}\right]$ = 1 mM, [HRP] = 0.5 µM, *E-E_h_* = −0.35 V.

To help validate the mechanistic model, trends in the model’s prediction of how each of the four independent variables influenced the EI’s signal were compared to the corresponding experimental data (Figures 5–8). The strength of each independent variable’s effect was quantified as the standardized effect (SE) value [42] in the Pareto chart (Figure 4) generated by Minitab^®^. The dotted line marks the minimum SE value for statistical significance at the 95% confidence level (SE = 2.09). These results indicate that all four independent variables significantly affected the signal, with the strength of those effects decreasing in the order *E* (SE = 11.4) > [*C*] (SE = 8.9) > pH (SE = 4.6) > [$H_{2}O_{2}$] (SE = 2.1).

The strong increase in the EI’s signal with *E*, and thus the magnitude of (*E-E_h_*), is apparent in both the experimental results and the model’s predictions (Figure 5). This trend is attributed to the Butler–Volmer Equation’s (2) exponential dependency of the EI’s amperometric signal on (*E-E_h_*).

The effects of the two HRP substrate concentrations, *C* and $H_{2}O_{2}$, predicted by the model are also similar to those observed experimentally (Figure 6 and Figure 7, respectively). The increase in signal with an increase in each substrate’s concentration, is consistent with the ping-pong kinetic model (Equation (1)), which predicts that HRP’s reaction rate would increase as either *C* or $H_{2}O_{2}$ increases. However, the SE for *C* is considerably stronger (SE = 8.9) than that for $H_{2}O_{2}$ (SE = 2.1), possibly because the $H_{2}O_{2}$ used in the experiments was much greater than the $K_{m}^{H_{2}O_{2}}$ value for HRP.

Both the experimental results and the mechanistic model (Figure 8) indicated a slightly higher EI signal in a mildly acidic bulk solution (pH = 6.2 or 6.6) than a neutral one (pH = 7). This trend is consistent with published reports that HRP oxidized substrates more rapidly in slightly acidic buffer than in neutral buffer [57]. One explanation for this effect is that pH (i.e., proton concentration) affects the thermodynamic driving force for the two-electron, two-proton electrochemical reduction of *Q* to *C* at the electrode. The $E_{h}$ value used in the model was measured as the midpoint potential ($E_{mid}$) of a cyclic voltammograms of an aqueous solution containing *C* and *Q*. Equation (3) shows that increasing pH would make $E_{mid}$ more negative, which would reduce the magnitude of (*E-E_h_*)and thereby reduce the EI’s signal [43,44].

### 4.3. Integration of Dimensional Analysis and Flux Analysis to Determine Rate-Limiting Step

**Figure 9:**
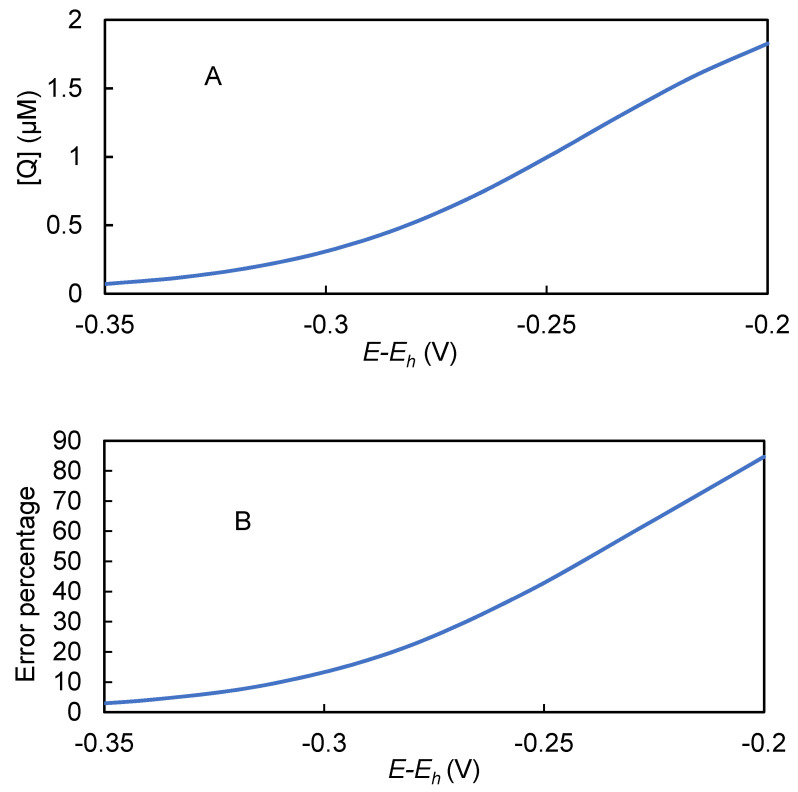
Simulated$\;{\left[Q\right]}_{x=0}$. (**A**): Simulated ${\left[Q\right]}_{x=0}$ over a range of (*E-E_h_*) values (**B**): Error percentage caused by assuming ${\left[Q\right]}_{x=0}$ = 0 as a function of (*E-E_h_*). Error percentage = [(*J* assuming ${\left[Q\right]}_{x=0}$ = 0–*J* using calculated value of ${\left[Q\right]}_{x=0}$ )/*J* using calculated value of ${\left[Q\right]}_{x=0}$ ] * 100. [C] = 8 mM, $\left[H_{2}O_{2}\right]$ = 1 mM, pH = 6.2, [HRP] = 0.5 µM.

Previous mathematical models developed to describe kinetics of HRP on the electrodes [21,37,38,39,40] focused on the enzyme’s kinetics or were based on an assumption that the *J* is mass-transfer limited. In contrast, our model explicitly calculates the rates of all key reaction and mass transfer steps, all of which could limit the signal’s magnitude to some extent. Additionally, incorporation of Equations (2) and (3) allows effects of (*E-E_h_*) and pH, respectively, to be predicted, even under conditions in which the commonly used assumption that ${\left[Q\right]}_{x=0}$ = 0 is invalid. Figure 9A shows that ${\left[Q\right]}_{x=0}$ decreases as the magnitude of (*E-E_h_*) and the reduction rate of [*Q*] increases.

This extension of the model provides a significant improvement in accuracy over models based on the commonly used assumption that ${\left[Q\right]}_{x=0}\;$= 0. Figure 9B shows the percent error in predicted output current caused by that assumption under the experimentally realistic range of (*E-E_h_*) values between −0.2 and −0.35 V. Notably, the error would have been about 15% for the (*E-E_h_*) value of −0.3 V used by Kohli et al. [47,58], whose model was based on the assumption that that ${\left[Q\right]}_{x=0}$ = 0. A strategy of reducing the magnitude of the overpotential to reduce the modeling error due to that assumption is likely to be counterproductive, because that strategy would also reduce the biosensor’s signal.

The performance properties of an EI are controlled by the dynamics of the underlying transport and reaction steps that give rise to its *J*. We developed a mathematical framework that leverages dimensional analysis and the mechanistic model’s ability to predict the rates of the underlying steps to quantitatively assess the degree to which individual steps control the magnitude of the EI’s signal and its sensitivity (defined as change in *J* per unit change in analyte concentration). Examples of the approach are described below.

The dimensionless Damkohler number (σ) shown in Equation (17) expresses the ratio of the relative rates of enzymatic reaction ($\frac{V_{max}}{K_{M}}$) and diffusional mass transfer ($\frac{D_{L}}{L^{2}})$ of HRP’s substrates within the immunosensing layer [59]. Plugging constants from Table 1 into Equation (17) revealed that σ for *C* and $H_{2}O_{2}$ were on the order of 10^−5^, indicating that the diffusion could provide *C* and $H_{2}O_{2}$ to the HRP orders of magnitude faster than the HRP could consume it [60,61]. This result indicates that the EI’s signal is not significantly limited by the diffusion rate within the immunosensing layer.

(17) $$\sigma^{2}=\frac{V_{max}L^{2}}{D_{L}K_{M}}$$

Flux-control analysis has been used to determine the extent to which the rates of individual enzymatic reactions in a biochemical reaction pathway limit the overall mass flux through that pathway [62]. We used a similar approach to determine the relative degrees to which the enzymatic and electrochemical reaction steps limit the magnitude of EI’s signal. We defined a current-control coefficient ($C_{Vi}^{J})\;$for each reaction step (*V_i_*) as the ratio of the percent change in the EI’s signal to the percent change in V_i_ while holding all other independent variables constant Equation (18). We used the mechanistic model to calculate an incremental change in *J* (∆*J*) resulting from an incremental change (∆$V_{i}$) in either the enzymatic reaction rate (simulated by changing the [HRP] value) or the electrochemical reaction rate (simulated by changing the (*E-E_h_*) value). The incremental changes (∆J and ∆$V_{i})$ were then used in place of the differentials (∂J and $\partial V_{i}$) in Equation (18) to calculate the $C_{Vi}^{J}$ values for both the enzymatic reaction and the electrochemical reaction across the range of (*E-E_h_*) values used in this study.

(18) ∂JJ∂ViVi=CViJ

The $C_{Vi}^{J}$ values calculated by making incremental changes in [HRP] remained virtually 1.0 across the entire range of (*E-E_h_*), for the HRP value listed in Table 1 (0.5 μm), as well as values ranging from 0.005 µM to 50 µM (results not shown). This result indicates that the EI’s signal is strongly limited by [HRP] over the entire range simulated. Consequently, the EI’s signal has the potential to be linearly correlated with the target analyte’s concentration, depending on the shape of the adsorption isotherm of the immobilized primary antibody for its target analyte.

**Figure 10:**
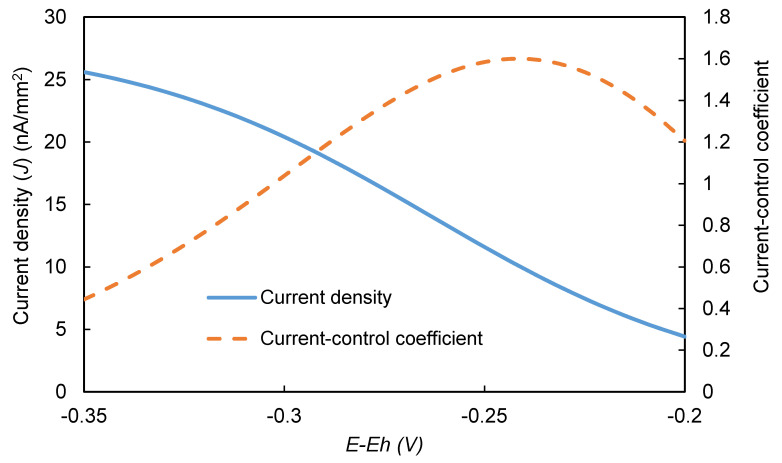
Predicted current density (*J*) and current-control coefficients for the electrochemical reaction at different *E* values. [*C*] = 8 mM, $\left[H_{2}O_{2}\right]$ = 1 mM, pH = 6.2, [HRP] = 0.5 µM.

In contrast, the $C_{Vi}^{J}$ values for the electrochemical reaction varied significantly across the range of overpotential used in this study (Figure 10) and exhibited a peak at about 1.5 at an (*E-E_h_*) value of about −0.24 V. Although the predicted EI’s signal curve increased monotonically as the magnitude of (*E-E_h_*) increased, the curve exhibited an inflection point at about the same (*E-E_h_*) value the $C_{Vi}^{J}$ curve peaked. This observation suggests that a transition occurs at this point. For lower (*E-E_h_*) magnitudes, increasing the magnitude strongly increases the EI’s signal. However, for higher (*E-E_h_*) magnitudes, further increases in the (*E-E_h_*) magnitude offer diminishing returns, suggesting that the peak in $C_{Vi}^{S}$ may mark an optimal operating overpotential in the absence of other overriding considerations, such as the presence of electrochemical interferents. For significantly higher (*E-E_h_*) magnitudes, the *J* asymptotically approaches a maximum value, and the $C_{Vi}^{J}$ value approaches 0 (Supplementary Figure S3).

Because [HRP] would be expected to increase with the analyte concentration, the mechanistic model was also used to calculate the EI’s sensitivity (*S*) to HRP (defined in Equation (19)), as well as sensitivity-control coefficients ($C_{Vi}^{S})$ (defined in Equation (20)).

(19) $$S≃\frac{dJ}{d\left[HRP\right]}$$

(20) ∂SS∂ViVi=CViS

**Figure 11:**
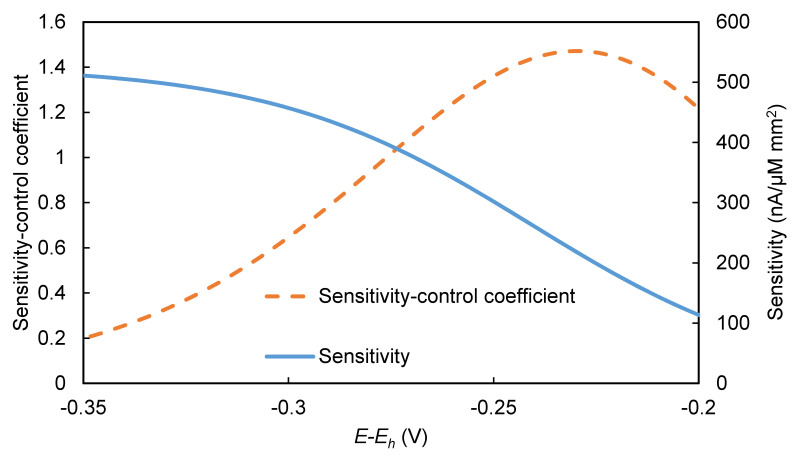
Sensitivity-control coefficient and sensitivity vs. *E-E_h_*. [*C*] = 8 mM, $\left[H_{2}O_{2}\right]$ = 1.0 mM, pH = 6.2, [HRP] = 0.5 µM.

The *S* and $C_{Vi}^{S}$ values were calculated in a manner similar to that used to calculate $C_{Vi}^{J}$ values. The model was used to calculate incremental ∆*J* values resulting from incremental ∆[HRP] values. The incremental change values were substituted for differentials in Equations (19) and (20). The resulting *S* values and $C_{Vi}^{S}$ values (Figure 11) have shapes similar to the *J* and $C_{Vi}^{J}$ curves, respectively, shown in Figure 10. However, the peak in the $C_{Vi}^{S}$ curve occurs at a slightly different (*E-E_h_*) value (−0.23 V) than the peak in the $C_{Vi}^{J}$ curve (−0.26 V). If an EI were operated near the peak of the $C_{Vi}^{S}$ curve, the sensitivity could be adjusted simply by making a relatively small change in the (*E-E_h_*) value. The ability to adjust an EI’s sensitivity may provide users some flexibility to tailor the tradeoff between precision and usable analyte concentration range. Assuming an EI can only be used over a fixed range of amperometric signals, a higher sensitivity would be expected to provide higher precision but a narrower useful concentration range, whereas a lower sensitivity would be expected to provide lower precision but a wider useful concentration range.

Our mechanistic model could readily be adapted to other systems and used for other purposes than the ones shown here. For example, Kergaravat et al. optimized conditions for electrochemically measuring HRP’s oxidation rate for seven redox-active co-substrates [63]. For each co-substrate, they optimized the pH and the concentrations of HRP, $H_{2}O_{2}$, and the co-substrate. They also reported the measured current as a function of working electrode potential for each co-substrate. Our model could be fit to their experimental data by adjusting parameters, such as HRP’s kinetic constants and the midpoint potential for each co-substrate. In addition, the effect of pH on the electrochemical reduction of the oxidized co-substrate could be modeled using the Butler–Volmer Equation (2). The authors also reported dynamics of the HRP reaction (current vs. time), both to monitor the batch reaction after the co-substrate or $H_{2}O_{2}$ was added to the reaction mixture, and to monitor the rate of change in current following a change in the working electrode’s potential [63]. These results could be simulated by adding an accumulation term (i.e., a time derivative) for the chemical species being balanced on the left-hand side of Equations (4)–(6). The resulting system of partial differential equations could be solved to generate the types of time-dependent curves described above.

Our model could be adapted to describe other reporter enzymes that can be coupled to redox-active products that could be reversibly oxidized and reduced. For example, the commonly used reporter alkaline phosphatase (AP) [64] can hydrolyze phenylphosphate to phenol, which can then be oxidized to O-quinone by tryrosinase [65] and then measured electrochemically via reduction at an electrode [66,67,68]. The model could also be adapted to other redox-active phenolic or aromatic co-substrates of HRP, including o-phenylenediamine [18], 3,3′,5,5′-tertramethyl benzidine (TMB) [69], and *p*-aminophenol (PAP) [70]. Each co-substrate’s electrochemical reduction kinetics could be accurately described by substituting appropriate *E_h_* and $K_{0}$ values into the model.

Another natural extension of the model would be to add equations that describe the equilibrium partitioning of the antigen binding to the immobilized primary antibody and the labeled secondary binding to the antigen-primary-antibody complex to form the sandwich molecular structure. This extension would allow the concentration of reporter enzyme (e.g., HRP) bound to the electrode, and thus allow the current generated by the EI to be predicted as a function of the antigen concentration in the sample. This capability would be useful for designing EI systems that meet desired performance specifications.

The novel dimensionless groups defined in this paper will enable future EI systems to be designed to meet performance specifications. For example, for an EI to have high sensitivity, it should be designed such that the CCC for the labeling enzyme is close to 1, indicating that that enzyme is highly rate-limiting. Also, an EI could be designed so that it’s sensitivity gives the desired balance between the precision and breadth of the useful analyte concentration range. Assuming an EI can only be used over a fixed range of amperometric signal, a higher sensitivity would be expected to provide higher precision but a narrower useful concentration range, whereas a lower sensitivity would be expected to provide lower precision but a wider useful concentration range.

Furthermore, our model could be extended to simulate some nonideal electrochemical effects. For example, when using cyclic voltammetry to determine the *E_h_* value, the voltage difference between the oxidation and reduction peaks (i.e., the peak separation) can vary significantly with experimental conditions. A minimum peak separation of about 60 mV/n would be expected for an ideal system (rapid diffusion and a rapid, reversible redox reaction). However, a larger peak separation would be expected for slower electron-transfer kinetics and/or slower diffusion [71,72]. Thus, variations in peak separation could, in principle, be simulated by adjusting the model’s $D_{L}$ and $K_{0}$ values.

## 5. Conclusions

This study demonstrated the use of a novel, integrated experimental and modeling framework to analyze and optimize the performance of EIs. The experimental component included (1) deposition of an EI interface on the working electrode of miniature SPE arrays; (2) measurement of the performance properties of the resulting EIs for measuring the concentration of a surrogate protein antigen (mouse IgG); (3) use of a response-surface, statistical-design-of-experiments approach to optimize four independent variables: electrode overpotential, pH, and the concentrations of HRP’s two substrates ([*C*] and $\left[H_{2}O_{2}\right]$); and (4) development of a statistical model of the experimental data that empirically describes the effect of the four independent variables on the EI’s signal.

The modeling component included (1) development of a detailed, mechanistic model of the EI interface that described the rates of the mass-transfer and reaction steps that gave rise to the EI’s signal; (2) use of the statistical model of the experimental data to help validate the mechanistic model; and (3) integration of dimensional analysis, principles of flux-control analysis, and the mechanistic model’s predictive capabilities to obtain unprecedented insight into which steps control the magnitude of the EI’s signal and its sensitivity to the target analyte.

The EI developed in this study had a limit of detection of 1 ng/mL, and an inter-assay/intra-assay variation of less than 5%. The mechanistic model was able to reproduce experimentally observed effects of the four independent variables on the EI’s signal. Calculation of Damkohler numbers indicated that diffusion of HRP’s substrates in the biocatalytic layer did not limit the EI’s performance at the overpotential of −0.3 V. Calculation of current-control and sensitivity-control coefficients analyses provided new insight into the extent to which the enzymatic and electrochemical reactions limited both the EI’s signal and its sensitivity over the experimentally relevant range of (*E-E_h_*) values.

The novel, integrated experimental and modeling framework presented in this study provides unprecedented capabilities to design, optimize, and validate EIs for diverse applications. Its ability to quickly identify key mass transfer or reaction step(s) that limit(s) could guide strategies to overcome such limitation(s) and thereby reduce time required to develop new commercial EI systems. Also, the predictive power of the mechanistic model could, in principle, enable EIs to be designed a priori to meet specifications and enable rapid, in-silico hypothesis testing that could accelerate FDA approval of EI systems for healthcare applications.

## Figures and Tables

**Table 1:** Values of constants used in the mechanistic model. The $K_{0}$ and [HRP] values were fit to the experimental data obtained using a constant analyte concentration of 40 ng/mL.

Parameter	Value	Units	Source
$k^{cat}$	2.2 × 10^4^	s^−1^	[46]
[HRP]	0.5	µM	-
$K_{m}^{C}$	3.0	mM	[46]
$K_{m}^{H_{2}O_{2}}$	0.2	mM	[46]
$K_{0}$	8.0 × 10^−7^	cm s^−1^	-
$E_{h}$	0.15	V	-
*δ*	3.0 × 10^−3^	cm	[55]
*L*	25	nm	-
$D_{\delta }$	2.2 × 10^−5^	cm^2^ s^−1^	-
$D_{L}$	2.3 × 10^−6^	cm^2^ s^−1^	-
$k_{p}$	1.0	-	-

## Supplementary Materials

The following are available online at https://www.mdpi.com/2079-6374/10/10/144/s1, Figure S1: Control experiment to measure background current caused by addition of 1 mM H_2_O_2_ at (*E-E_h_*) of −0.35 V. Figure S2. The dose response for mouse IgG on gold Dropsens SPEs before ($\left[H_{2}O_{2}\right]$ = 1.5 mM, pH = 7, [C] = 7 mM, *E-E_h_* = −0.3 V) and after optimization ($\left[H_{2}O_{2}\right]\;$= 1 mM, pH = 6.2, [C] = 8 mM, *E-E_h_* = −0.35 V). Error bars show ± standard deviation from the mean of 3 replicates. Figure S3. Predicted current density and current-control coefficients for the electrochemical reaction at different E values. [C] = 8 mM, $\left[H_{2}O_{2}\right]$ = 1 mM, pH = 6.2, [HRP] = 5 µM. Table S1: Design of experiments in coded units suggested by MINITAB using half-factorial design, MATLAB codes for generation of mechanistic model’s results in Figure 5, Figure 6, Figure 7 and Figure 8.

## References

[1] Ricci F., Adornetto G., Palleschi G. (2012). A review of experimental aspects of electrochemical immunosensors. Electrochim. Acta.

[2] Wang J. (2008). ChemInform Abstract: Electrochemical Glucose Biosensors. Chem. Rev..

[3] Heinemann L., Klonoff D.C. (2013). Blood Glucose Meter Market: This World is Undergoing Drastic Changes. J. Diabetes Sci. Technol..

[4] Kang M.-H., Kim D.-H., Jeong I.-S., Choi G.-C., Park H.-M. (2015). Evaluation of four portable blood glucose meters in diabetic and non-diabetic dogs and cats. Veter Q..

[5] Chen A., Yang S. (2015). Replacing antibodies with aptamers in lateral flow immunoassay. Biosens. Bioelectron..

[6] Jiang N., Ahmed R., Damayantharan M., Ünal B., Butt H., Yetisen A.K. (2019). Lateral and Vertical Flow Assays for Point-of-Care Diagnostics. Adv. Heal. Mater..

[7] Wu J., Fu Z., Yan F., Ju H. (2007). Biomedical and clinical applications of immunoassays and immunosensors for tumor markers. TrAC Trends Anal. Chem..

[8] Wen W., Yan X., Zhu C., Du D., Lin Y. (2016). Recent Advances in Electrochemical Immunosensors. Anal. Chem..

[9] Abuknesha R., Luk C.Y., Griffith H.H., Maragkou A., Iakovaki D. (2005). Efficient labelling of antibodies with horseradish peroxidase using cyanuric chloride. J. Immunol. Methods.

[10] Ronkainen N.J., Halsall H.B., Heineman W.R. (2010). Electrochemical biosensors. Chem. Soc. Rev..

[11] Wan Y., Su Y., Zhu X., Liu G., Fan C. (2013). Development of electrochemical immunosensors towards point of care diagnostics. Biosens. Bioelectron..

[12] Bahadır E.B., Sezgintürk M.K. (2015). Applications of electrochemical immunosensors for early clinical diagnostics. Talanta.

[13] Ghindilis A.L., Atanasov P., Wilkins M., Wilkins E., Ghindilis A. (1998). Immunosensors: Electrochemical sensing and other engineering approaches. Biosens. Bioelectron..

[14] Skládal P. (1997). Advances in electrochemical immunosensors. Electroanal.

[15] Lisi F., Peterson J.R., Gooding J.J. (2020). The application of personal glucose meters as universal point-of-care diagnostic tools. Biosens. Bioelectron..

[16] Bahri M., Baraket A., Zine N., Ben Ali M., Bausells J., Errachid A. (2020). Capacitance electrochemical biosensor based on silicon nitride transducer for TNF-α cytokine detection in artificial human saliva: Heart failure (HF). Talanta.

[17] Tallapragada S.D., Layek K., Mukherjee R., Mistry K.K., Ghosh M. (2017). Development of screen-printed electrode based immunosensor for the detection of HER2 antigen in human serum samples. Bioelectrochemistry.

[18] Dempsey E., Rathod D. (2018). Disposable Printed Lateral Flow Electrochemical Immunosensors for Human Cardiac Troponin T. IEEE Sens. J..

[19] Kulys J., Baronas R. (2006). Modelling of Amperometric Biosensors in the Case of Substrate Inhibition. Sensors.

[20] Nicell J.A., Wright H. (1997). A model of peroxidase activity with inhibition by hydrogen peroxide. Enzym. Microb. Technol..

[21] Ruzgas T., Gorton L., Emnéus J., Marko-Varga G. (1995). Kinetic models of horseradish peroxidase action on a graphite electrode. J. Electroanal. Chem..

[22] Vojinovic V., Carvalho R., Lemos F., Cabral J.M., Da Fonseca L.J.P., Ferreira B.S. (2007). Kinetics of soluble and immobilized horseradish peroxidase-mediated oxidation of phenolic compounds. Biochem. Eng. J..

[23] Buchanan I.D., Nicell J.A. (1999). A simplified model of peroxidase-catalyzed phenol removal from aqueous solution. J. Chem. Technol. Biotechnol..

[24] Katz L.B., Stewart L., King D., Cameron H. (2020). Meeting the New FDA Standard for Accuracy of Self-Monitoring Blood Glucose Test Systems Intended for Home Use by Lay Users. J. Diabetes Sci. Technol..

[25] Raghav R., Srivastava S. (2016). Immobilization strategy for enhancing sensitivity of immunosensors: L -Asparagine–AuNPs as a promising alternative of EDC–NHS activated citrate–AuNPs for antibody immobilization. Biosens. Bioelectron..

[26] Duan C., Meyerhoff M.E. (1995). Immobilization of proteins on gold coated porous membranes via an activated self-assembled monolayer of thioctic acid. Microchim. Acta.

[27] Pei Z., Anderson H., Myrskog A., Dunér G., Ingemarsson B., Aastrup T. (2010). Optimizing immobilization on two-dimensional carboxyl surface: pH dependence of antibody orientation and antigen binding capacity. Anal. Biochem..

[28] Scientific T. Carbodiimide Crosslinker Chemistry. https://www.thermofisher.com/us/en/home/life-science/protein-biology/protein-biology-learning-center/protein-biology-resource-library/pierce-protein-methods/carbodiimide-crosslinker-chemistry.html.

[29] Alonso-Lomillo M.A., Kauffmann J., Martinez M.A., Arcos-Martínez M. (2003). HRP-based biosensor for monitoring rifampicin. Biosens. Bioelectron..

[30] Belluzo M.S., Ribone M.É., Lagier C.M. (2008). Assembling Amperometric Biosensors for Clinical Diagnostics. Sensors.

[31] Branch G.E.K., Joslyn M.A. (1935). The Kinetics of the Auto-oxidation of Catechol in the Presence of Several Foreign Substances. J. Am. Chem. Soc..

[32] Baronas D., Ivanauskas F., Baronas R. (2011). Mechanisms controlling the sensitivity of amperometric biosensors in flow injection analysis systems. J. Math. Chem..

[33] Loghambal S., Rajendran L. (2011). Mathematical modeling in amperometric oxidase enzyme–membrane electrodes. J. Membr. Sci..

[34] Choi Y.-J., Chae H.J., Kim E.Y. (1999). Steady-state oxidation model by horseradish peroxidase for the estimation of the non-inactivation zone in the enzymatic removal of pentachlorophenol. J. Biosci. Bioeng..

[35] Wu Y., Taylor K.E., Bewtra J.K., Biswas N. (1999). Kinetic Model for Removal of Phenol by Horseradish Peroxidase with PEG. J. Environ. Eng..

[36] Mansouri Majoumerd M., Kariminia H.R. (2013). Bisubstrate kinetic model for enzymatic decolorization of reactive black 5 by Coprinus cinereus Peroxidase. Iran. J. Chem. Chem. Eng. (IJCCE).

[37] Huang J., Huang W., Wang T. (2012). Catalytic and Inhibitory Kinetic Behavior of Horseradish Peroxidase on the Electrode Surface. Sensors.

[38] Ivanec-Goranina R., Kulys J. (2008). Kinetic study of peroxidase-catalyzed oxidation of 1-hydroxypyrene. Development of a nanomolar hydrogen peroxide detection system. Open Life Sci..

[39] Galende P.P., Cuadrado N.H., Kostetsky E., Roig M.G., Villar E., Shnyrov V.L., Kennedy J.F. (2015). Kinetics of Spanish broom peroxidase obeys a Ping-Pong Bi–Bi mechanism with competitive inhibition by substrates. Int. J. Biol. Macromol..

[40] Deyhimi F., Nami F. (2012). Peroxidase-catalyzed electrochemical assay of hydrogen peroxide: A ping-pong mechanism. Int. J. Chem. Kinet..

[41] Šimelevicius D., Petrauskas K. Application of the Butler-Volmer Equation in Mathematical Modelling of Amperometric Biosensor. Proceedings of the Sixth International Conference on Advances in System Simulation.

[42] Compton R.G., Banks C.E. (2011). Understanding Voltammetry.

[43] Lin Q., Li Q., Batchelor-McAuley C., Compton R.G. (2015). Two-Electron, Two-Proton Oxidation of Catechol: Kinetics and Apparent Catalysis. J. Phys. Chem. C.

[44] Compton R.G., Wadhawan J. (2013). Electrochemistry: Nanoelectrochemistry.

[45] Guérente L., Desprez V., Diard J.-P., Labbé P. (1999). Amplification of amperometric biosensor responses by electrochemical substrate recycling: Part I. Theoretical treatment of the catechol–polyphenol oxidase system. J. Electroanal. Chem..

[46] Guérente L., Desprez V., Labbe P., Therias S. (1999). Amplification of amperometric biosensor responses by electrochemical substrate recycling: Part II. Experimental study of the catechol–polyphenol oxidase system immobilized in a laponite clay matrix. J. Electroanal. Chem..

[47] Guérente L., Labbé P., Mengeaud V. (2001). Amplification of amperometric biosensor responses by electrochemical substrate recycling. 3. Theoretical and experimental study of the phenol-polyphenol oxidase system immobilized in Laponite hydrogels and layer-by-layer self-assembled structures. Anal. Chem..

[48] Kohli N., Lee I., Richardson R.J., Worden R.M. (2010). Theoretical and experimental study of bi-enzyme electrodes with substrate recycling. J. Electroanal. Chem..

[49] Indira K., Lakshmanan R. (2012). Analytical Expressions Pertaining to the Concentration of Substrates and Product in Phenol-Polyphenol Oxidase System Immobilized in Laponite Hydrogels: A Reciprocal Competitive Inhibition Process. Adv. Phys. Chem..

[50] Achi F., Bourouina-Bacha S., Bourouina M., Amine A. (2015). Mathematical model and numerical simulation of inhibition based biosensor for the detection of Hg(II). Sens. Actuators B Chem..

[51] Meena A., Rajendran L. (2010). Mathematical modeling of amperometric and potentiometric biosensors and system of non-linear equations—Homotopy perturbation approach. J. Electroanal. Chem..

[52] BRENDA The Comprehensive Enzyme Information System. https://www.brenda-enzymes.org/.

[53] Amatore C., Szunerits S., Thouin L., Workocz J.-S. (2001). The real meaning of Nernst’s steady diffusion layer concept under non-forced hydrodynamic conditions. A simple model based on Levich’s seminal view of convection. J. Electroanal. Chem..

[54] Fransaer J., Ammam M., Jan F., Ammam M. (2011). Mathematical Modeling of the Amperometric Response to Glucose of Glucose Oxidase Films Deposited by AC-Electrophoresis. J. Sens. Technol..

[55] Ašeris V., Gaidamauskaitė E., Kulys J., Baronas R. (2014). Modelling glucose dehydrogenase-based amperometric biosensor utilizing synergistic substrates conversion. Electrochim. Acta.

[56] Grünwald P. (1989). Determination of effective diffusion coefficients—An important parameters for the efficiency of immobilized biocatalysts. Biochem. Educ..

[57] Ximenes V.F., Fernandes J.R., Bueno V.B., Catalani L.H., De Oliveira G.H., Machado R.G.P. (2007). The effect of pH on horseradish peroxidase-catalyzed oxidation of melatonin: Production of N1-acetyl-N2-formyl-5-methoxykynuramine versus radical-mediated degradation. J. Pineal Res..

[58] Kohli N., Srivastava D., Sun J., Richardson R.J., Lee I., Worden R.M. (2007). Nanostructured Biosensor for Measuring Neuropathy Target Esterase Activity. Anal. Chem..

[59] Parthasarathy P., Vivekanandan S. (2018). A numerical modelling of an amperometric-enzymatic based uric acid biosensor for GOUT arthritis diseases. Inform. Med. Unlocked.

[60] Ismail I., Oluleye G., Oluwafemi I. (2017). Mathematical modelling of an enzyme-based biosensor. Int. J. Biosens. Bioelectron..

[61] Baronas R., Kulys J., Lančinskas A., Žilinskas A. (2014). Effect of Diffusion Limitations on Multianalyte Determination from Biased Biosensor Response. Sensors.

[62] Kacser H., Burns J.A., Fell D.A. (1995). The Control of Flux.

[63] Kergaravat S.V., Pividori M.I., Hernandez S.R. (2012). Evaluation of seven cosubstrates in the quantification of horseradish peroxidase enzyme by square wave voltammetry. Talanta.

[64] Mistry K.K., Layek K., Mahapatra A., Roychaudhuri C., Saha H. (2014). A review on amperometric-type immunosensors based on screen-printed electrodes. Analyst.

[65] Akanda R., Ju H. (2016). A Tyrosinase-Responsive Nonenzymatic Redox Cycling for Amplified Electrochemical Immunosensing of Protein. Anal. Chem..

[66] Kohli N., Srivastava D., Sun J., Richardson R.J., Lee I., Worden R.M. (2014). Nanostructured Biosensor Containing Neuropathy Target Esterase Activit. U.S. Patent.

[67] Carralero V., González-Cortés A., Yáñez-Sedeño P., Pingarrón J. (2007). Nanostructured progesterone immunosensor using a tyrosinase–colloidal gold–graphite–Teflon biosensor as amperometric transducer. Anal. Chim. Acta.

[68] Escamilla-Gómez V., Campuzano S., Pedrero M., Pingarrón J. (2008). Immunosensor for the determination of Staphylococcus aureus using a tyrosinase–mercaptopropionic acid modified electrode as an amperometric transducer. Anal. Bioanal. Chem..

[69] Ahirwal G.K., Mitra C.K. (2010). Gold nanoparticles based sandwich electrochemical immunosensor. Biosens. Bioelectron..

[70] Sun W., Jiao K., Zhang S., Zhang C., Zhang Z. (2001). Electrochemical detection for horseradish peroxidase-based enzyme immunoassay using p-aminophenol as substrate and its application in detection of plant virus. Anal. Chim. Acta.

[71] Elgrishi N., Rountree K.J., McCarthy B.D., Rountree E.S., Eisenhart T.T., Dempsey J.L. (2017). A Practical Beginner’s Guide to Cyclic Voltammetry. J. Chem. Educ..

[72] Savéant J.-M. (2006). Elements of Molecular and Biomolecular Electrochemistry.

