# Supplementary material for: Integrated Experimental and Theoretical Studies on an Electrochemical Immunosensor
Source: Biosensors (Basel). 2020 Oct 17;10(10):144. doi: 10.3390/bios10100144 (PMC7603011; doi:10.3390/bios10100144)
Supplement: Supplementary file 1 [file biosensors-10-00144-s001.pdf]

Table S1. Design of Experiments in coded units suggested by MINITAB using half factorial design.

Run Order	E	pH	C	H_2O_2	Run Order	E	pH	C	H_2O_2
1	+1	-1	-1	+1	19	0	0	0	0
2	-1	+1	+1	-1	20	-1	-1	-1	-1
3	0	0	0	0	21	+1	+1	+1	+1
4	0	0	0	0	22	0	0	0	0
5	+1	-1	+1	-1	23	-1	+1	-1	+1
6	-1	+1	-1	+1	24	+1	-1	+1	-1
7	-1	-1	+1	+1	25	-1	+1	+1	-1
8	+1	+1	-1	-1	26	+1	-1	-1	+1
9	0	0	0	0	27	0	0	0	0
10	-1	-1	+1	+1	28	0	0	0	0
11	0	0	0	0	29	-1	+1	+1	-1
12	+1	+1	-1	-1	30	+1	-1	-1	+1
13	0	0	0	0	31	-1	+1	-1	+1
14	+1	+1	+1	+1	32	0	0	0	0
15	-1	-1	-1	-1	33	+1	-1	+1	-1
16	+1	+1	+1	+1	34	+1	+1	-1	-1
17	0	0	0	0	35	0	0	0	0
18	-1	-1	-1	-1	36	-1	-1	+1	+1

The Eq S1, regression equation in coded units (-1, 0, or +1), was obtained from Minitab after analyzing factorial design. For each factor, three levels, denoted low (-1), center point (0), and high (+1), were selected: -0.05 V, -0.125 V, and -0.2 V for E; 1.0 mM, 4.5 mM, and 9.0 mM for $[C]$; 0.5 mM, 1mM, and 1.5 mM for $[H_2O_2]$; and 6.2, 6.6, and 7.0 for pH, respectively. Eq S1 returns the signal at $[HRP]$=0.5 µM when the parameters are plugged in the formula in their coded units (-1, 0, or +1).

$$\text{Signal} = 9.607 - 5.206\,E - 2.194\,pH + 4.072\,C + 1.031\,H_2O_2 + 1.951\,E*pH - 2.193*E*C - 1.759*E*H_2O_2 - 1.937*E*pH*C*H_2O_2 \qquad \text{Eq (S1)}$$

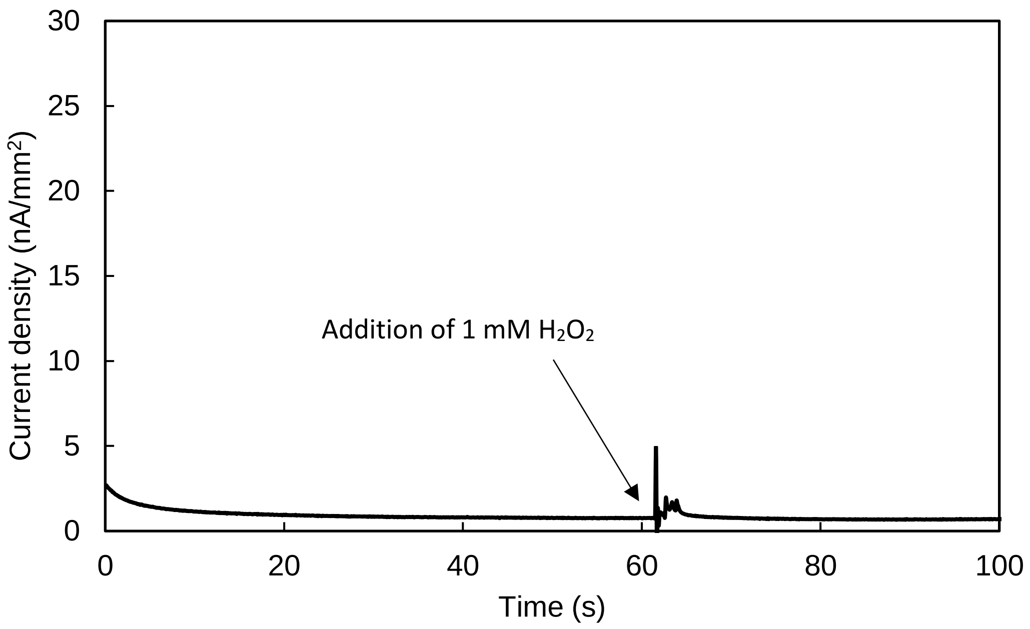

Figure S1. Control experiment to measure background current caused by addition of 1 mM H_2O_2 at (E-E_h) of -0.35 V. Steady-state current density after addition of H_2O_2 is 0.77 nA/mm^2.

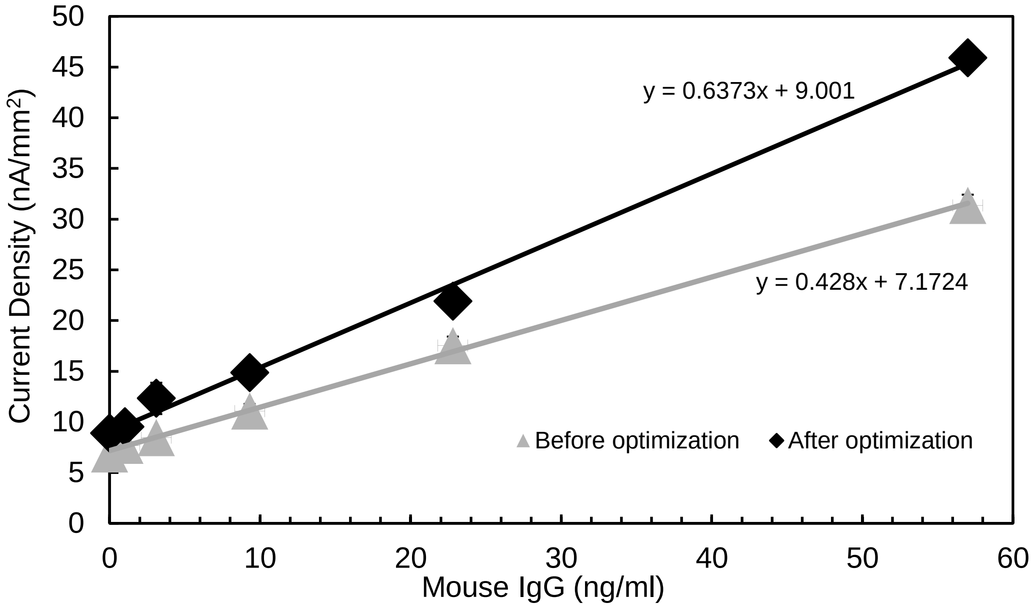

Figure S2. The dose response for mouse IgG on gold Dropsens SPEs before ($[H_2O_2]$=1.5 mM, pH=7 , [C] = 7 mM, E-E_h = -0.3 V) and after optimization ($[H_2O_2]$=1 mM, pH=6.2, [C] = 8 mM, E-E_h = -0.35 V). Error bars show ± standard deviation from the mean of 3 replicates.

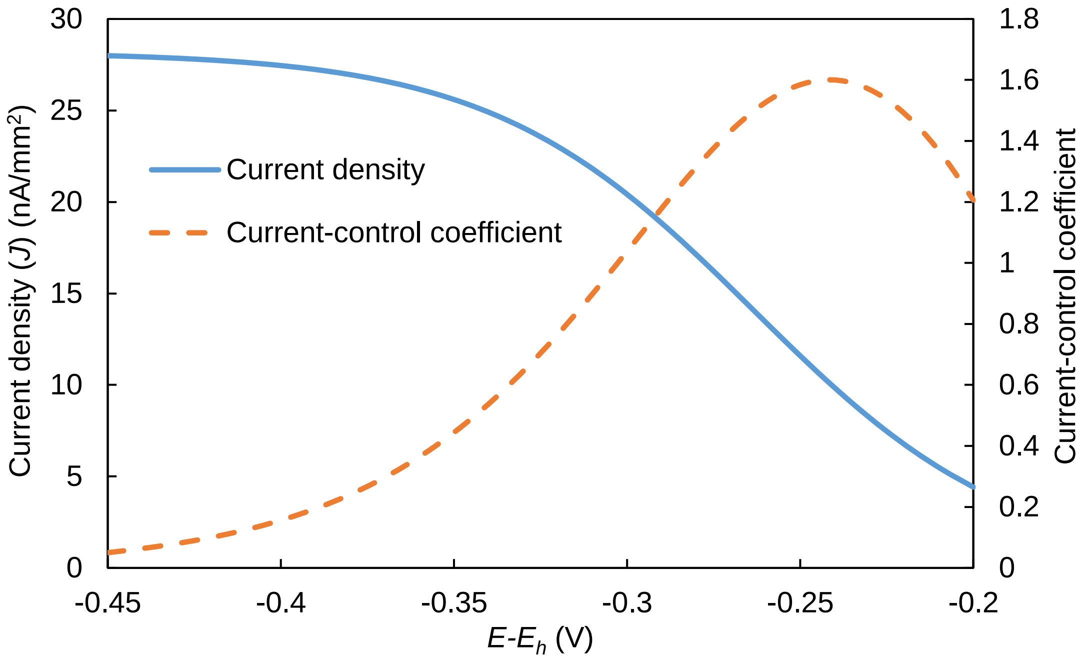

Figure S3. Predicted current density and current-control coefficients for the electrochemical reaction at different E values. [C]=8mM, [H_2O_2] =1 mM, pH=6.2, [HRP]=5μM.

MATLAB codes for generation of mechanistic model's results in Figures 5-8:

Appendix A

```
%%%% This function returns the effect of overpotential on EI's signal%%%%
function overpotential
H=1e-6; %H2O2 bulk concentration in mol/cm3
C=8e-6; %Catechol bulk concentration in mol/cm3
KHm=2e-7; %Km value of HRP for H2O2 in mol/cm3
KCm=3e-6; %Km value of HRP for catechol in mol/cm3
Kcat=22000; %turnover number of HRP for catechol and H2O2 in 1/sec
E=5e-10; %concentration of HRP in mol/cm3
L= 2.2e-6; %thickness of enzyme layer in cm
Df= 2.28e-6; %cm2/s %diffusion coefficient in enzyme layer
De= 2.2e-5; %cm2/s %diffusion coefficient in boundary layer
Kp= 1; %partition coefficient
del = 3e-3; %thickness of boundary layer in cm
Ka=0.1e-6; %apparent electron transfer rate in cm/s
R=8.314; %universal gas constant (8.314 J K-1 mol-1
T=298; %temperature in K
area= 0.118; %area of the working electrode in cm2
electron= 2; %number of electron transferred in reduction of quinone
F= 96485 %Faraday constant 96,485 C mol-1
x = linspace(0,L,100);
function dydx = ode3(x,y) %this function returns all of the odes, concentrations have been normalized by catechol bulk
concentration
dy1dx = [ y(2); (Kcat*E*y(1)*y(3)/(Df*(((KHm*KCm)/C)+KCm*y(3)+KHm*y(1)+(y(1)*y(3)*C))))];
dy2dx = [ y(4); (Kcat*E*y(1)*y(3)/(Df*(((KHm*KCm)/C)+KCm*y(3)+KHm*y(1)+(y(1)*y(3)*C))))];
dy3dx = [ y(6); -(Kcat*E*y(1)*y(3)/(Df*(((KHm*KCm)/C)+KCm*y(3)+KHm*y(1)+(y(1)*y(3)*C))))];
dydx=[dy1dx;dy2dx;dy3dx];
end

function res = ode3bc(ya,yb) %this function returns the BCs
res1 = [ya(4); Df*yb(2)-((De/(Kp*del))*(Kp-yb(1)))];%
```

```
res2 = [ya(2)+ya(6); Df*yb(4)-((De/(Kp*del))*(Kp*H/C-yb(3)))];%
res3 = [ya(6)-((((ya(5)*Ka*Exp)-(ya(3)*Ka*EXP))/(area*Df))); Df*yb(6)+((De/(Kp*del))*yb(5))];
res=[res1;res2;res3];
end
S=linspace(-0.05,-0.25,10);%E(applied voltage range)
i=1;
for i=1:length(S)
  V=S(i);
  Exp=exp((-F*0.8*((V-0.15)))/(R*T));%corresponds to butler-volmer
  EXP=exp((F*1.2*((V-0.15)))/(R*T));%corresponds to butler-volmer
  eexp(i)=Exp;
initialsolution = bvpinit(x,[1,0.001,1,-0.5,0.01,-0.06]);%initial guess
solution = bvp4c(@ode3,@ode3bc,initialsolution);
y = deval(solution,x);
D(i)= y(6,1);
J(i)=2*96485*Df*D(i)*C*(10000000); %current density in nA/mm2
end
figure (1)
hold on
plot(S,J);
xlabel('Potential(V)');
ylabel('Current density(nA/mm2)');
end
%%%%%%%%%%%%%%%%%%%%%%%%%%%%%%%%%%%%%%%%%%%%%%

%%%% This function returns the effect of pH on EI's signal%%%%
function hydrogenperoxide
H=1e-6; %H2O2 bulk concentration in mol/cm3
C=8e-6; %Catechol bulk concentration in mol/cm3
KHm=2e-7; %Km value of HRP for H2O2 in mol/cm3
KCm=3e-6; %Km value of HRP for catechol in mol/cm3
Kcat=22000; %turnover number of HRP for catechol and H2O2 in 1/sec
E=5e-10; %concentration of HRP in mol/cm3
L= 2.2e-6; %thickness of enzyme layer in cm
Df= 2.28e-6; %cm2/s %diffusion coefficient in enzyme layer
De= 2.2e-5; %cm2/s %diffusion coefficient in boundary layer
```

```
Kp= 1; %partition coefficient
del = 3e-3; %thickness of boundary layer in cm
Ka=0.1e-6; %apparent electron transfer rate in cm/s
R=8.314; %universal gas constant (8.314 J K-1 mol-1
T=298; %temperature in K
area= 0.118; %area of the working electrode in cm2
electron= 2; %number of electron transferred in reduction of quinone
F= 96485 %Faraday constant 96,485 C mol-1
V=-0.2; %applied voltage
   Exp=exp((-F*0.8*((V-0.15)))/(R*T));%corresponds to butler-volmer
   EXP=exp((F*1.2*((V-0.15)))/(R*T));%corresponds to butler-volmer
x = linspace(0,L,100);
function dydx = ode3(x,y) %this function returns all of the odes, concentrations have been normalized by catechol bulk concentration
dy1dx = [ y(2); (Kcat*E*y(1)*y(3)/(Df*(((KHm*KCm)/C)+KCm*y(3)+KHm*y(1)+(y(1)*y(3)*C))))];
dy2dx = [ y(4); (Kcat*E*y(1)*y(3)/(Df*(((KHm*KCm)/C)+KCm*y(3)+KHm*y(1)+(y(1)*y(3)*C))))];
dy3dx = [ y(6); -(Kcat*E*y(1)*y(3)/(Df*(((KHm*KCm)/C)+KCm*y(3)+KHm*y(1)+(y(1)*y(3)*C))))];
dydx=[dy1dx;dy2dx;dy3dx];
end

function res = ode3bc(ya,yb) %this function returns BCs
res1 = [ya(4); Df*yb(2)-((De/(Kp*del))*(Kp-yb(1)))];%
res2 = [ya(2)+ya(6); Df*yb(4)-((De/(Kp*del))*(Kp*H/C-yb(3)))];%
res3 = [ya(6)-((((ya(5)*Ka*Exp)-(ya(3)*Ka*EXP))/(area*Df))); Df*yb(6)+((De/(Kp*del))*yb(5))];
res=[res1;res2;res3];
end
S=linspace(0.5e-6,1.5e-6,10); %H2O2 range
i=1;
for i=1:length(S)
H=S(i);
initialsolution = bvpinit(x,[1,0.001,1,0.05,0.001,0.06]);
solution = bvp4c(@ode3,@ode3bc,initialsolution);
y = deval(solution,x);
D(i)= y(6,1);
J(i)=2*96485*Df*D(i)*C*(10000000); %current density
end
```

```
figure (1)
hold on
plot(S,J);
xlabel('H2O2(mM)');
ylabel('Currentdensity(nA/mm2)');
end
%%%%%%%%%%%%%%%%%%%%%%%%%%%%%%%%%%%%%%%%%%%%%%%%%

%%%% This returns the effect of catechol concentration on the EI's signal%%%%
function catechol
H=1e-6; %H2O2 bulk concentration in mol/cm3
C=8e-6; %Catechol bulk concentration in mol/cm3
KHm=2e-7; %Km value of HRP for H2O2 in mol/cm3
KCm=3e-6; %Km value of HRP for catechol in mol/cm3
Kcat=22000; %turnover number of HRP for catechol and H2O2 in 1/sec
E=5e-10; %concentration of HRP in mol/cm3
L= 2.2e-6; %thickness of enzyme layer in cm
Df= 2.28e-6; %cm2/s %diffusion coefficient in enzyme layer
De= 2.2e-5; %cm2/s %diffusion coefficient in boundary layer
Kp= 1; %partition coefficient
del = 3e-3; %thickness of boundary layer in cm
Ka=0.1e-6; % apparent electron transfer rate in cm/s
R=8.314; %universal gas constant (8.314 J K-1 mol-1
T=298; %temperature in K
area= 0.118; %area of the working electrode in cm2
electron= 2; %number of electron transferred in reduction of quinone
F= 96485 %Faraday constant 96,485 C mol-1
V=-0.2; %applied voltage
    Exp=exp((-F*0.8*((V-0.15)))/(R*T));%corresponds to butler-volmer
    EXP=exp((F*1.2*((V-0.15)))/(R*T));%corresponds to butler-volmer
x = linspace(0,L,100);
function dydx = ode3(x,y) %this function returns all of the odes, concentrations have been normalized by catechol bulk concentration
dy1dx = [ y(2); (Kcat*E*y(1)*y(3)/(Df*(((KHm*KCm)/C)+KCm*y(3)+KHm*y(1)+(y(1)*y(3)*C))))];
dy2dx = [ y(4); (Kcat*E*y(1)*y(3)/(Df*(((KHm*KCm)/C)+KCm*y(3)+KHm*y(1)+(y(1)*y(3)*C))))];
```

```
dy3dx = [ y(6); -(Kcat*E*y(1)*y(3)/(Df*(((KHm*KCm)/C)+KCm*y(3)+KHm*y(1)+(y(1)*y(3)*C))))];
dydx=[dy1dx;dy2dx;dy3dx];
end

function res = ode3bc(ya,yb)%this function returns all of the BCs
res1 = [ya(4); Df*yb(2)-((De/(Kp*del))*(Kp-yb(1)))];%
res2 = [ya(2)+ya(6); Df*yb(4)-((De/(Kp*del))*(Kp*H/C-yb(3)))];%
res3 = [ya(6)-((((ya(5)*Ka*Exp)-(ya(3)*Ka*EXP))/(area*Df))); Df*yb(6)+((De/(Kp*del))*yb(5))];
res=[res1;res2;res3];
end
S=linspace(1e-6,8e-6,10); %range of catechol
i=1;
for i=1:length(S)
C=S(i);
initialsolution = bvpinit(x,[1,0.001,1,0.05,0.001,0.06]);
solution = bvp4c(@ode3,@ode3bc,initialsolution);
y = deval(solution,x);
D(i)= y(6,1);
J(i)=2*96485*Df*D(i)*C*(10000000); %Current density in nA/mm2
end
hold on
plot(S*1e6,J);
xlabel('Catechol(mM)');
ylabel('Current density(nA/mm2)');
end
%%%%%%%%%%%%%%%%%%%%%%%%%%%%%%%%%%%%%%%%

%%%% This function returns the effect of pH on EI's signal%%%%
function hydrogenperoxide
H=1e-6; %H2O2 bulk concentration in mol/cm3
C=8e-6; %Catechol bulk concentration in mol/cm3
KHm=2e-7; %Km value of HRP for H2O2 in mol/cm3
KCm=3e-6; %Km value of HRP for catechol in mol/cm3
Kcat=22000; %turnover number of HRP for catechol and H2O2 in 1/sec
E=5e-10; %concentration of HRP in mol/cm3
L= 2.2e-6; %thickness of enzyme layer in cm
```

```
Df= 2.28e-6; %cm2/s %diffusion coefficient in enzyme layer
De= 2.2e-5; %cm2/s %diffusion coefficient in boundary layer
Kp= 1; %partition coefficient
del = 3e-3; %thickness of boundary layer in cm
Ka=0.1e-6; %apperant electron transfer rate in cm/s
R=8.314; %universal gas constant (8.314 J K-1 mol-1
T=298; %temperature in K
area= 0.118; %area of the working electrode in cm2
electron= 2; %number of electron transferred in reduction of quinone
F= 96485 %Faraday constant 96,485 C mol-1
V=-0.2; %applied voltage
   Exp=exp((-F*0.8*((V-0.15)))/(R*T));%corresponds to butler-volmer
   EXP=exp((F*1.2*((V-0.15)))/(R*T));%corresponds to butler-volmer
x = linspace(0,L,100);
function dydx = ode3(x,y) %this function returns all of the odes, concentrations have been normalized by catechol bulk concentration
dy1dx = [ y(2); (Kcat*E*y(1)*y(3)/(Df*(((KHm*KCm)/C)+KCm*y(3)+KHm*y(1)+(y(1)*y(3)*C))))];
dy2dx = [ y(4); (Kcat*E*y(1)*y(3)/(Df*(((KHm*KCm)/C)+KCm*y(3)+KHm*y(1)+(y(1)*y(3)*C))))];
dy3dx = [ y(6); -(Kcat*E*y(1)*y(3)/(Df*(((KHm*KCm)/C)+KCm*y(3)+KHm*y(1)+(y(1)*y(3)*C))))];
dydx=[dy1dx;dy2dx;dy3dx];
end


function res = ode3bc(ya,yb) %this function returns BCs
res1 = [ya(4); Df*yb(2)-((De/(Kp*del))*(Kp-yb(1)))];%
res2 = [ya(2)+ya(6); Df*yb(4)-((De/(Kp*del))*(Kp*H/C-yb(3)))];%
res3 = [ya(6)-((((ya(5)*Ka*Exp)-(ya(3)*Ka*EXP))/(area*Df))); Df*yb(6)+((De/(Kp*del))*yb(5))];
res=[res1;res2;res3];
end
S=linspace(0.5e-6,1.5e-6,10); %H2O2 range
i=1;
for i=1:length(S)
H=S(i);
initialsolution = bvpinit(x,[1,0.001,1,0.05,0.001,0.06]);
solution = bvp4c(@ode3,@ode3bc,initialsolution);
y = deval(solution,x);
D(i)= y(6,1);
```

```
J(i)=2*96485*Df*D(i)*C*(10000000); %current density
end
figure (1)
hold on
plot(S*1e6,J);
xlabel('H2O2(mM)');
ylabel('Current density(nA/mm2)');
end
%%%%%%%%%%%%%%%%%%%%%%%%%%%%%%%%%%%%%%%%%%%%%%
```
